# Mitochondrial Dysfunction and Atherosclerosis: The Problem and the Search for Its Solution

**DOI:** 10.3390/biomedicines13040963

**Published:** 2025-04-15

**Authors:** Ganna Nevoit, Gediminas Jarusevicius, Maksim Potyazhenko, Ozar Mintser, Inga Arune Bumblyte, Alfonsas Vainoras

**Affiliations:** 1 Laboratory for Automatization of Cardiovascular Investigations, Cardiology Institute, Lithuanian University of Health Sciences, LT-44307 Kaunas, Lithuania; 2 Department of Internal Medicine and Emergency Medicine, Poltava State Medical University, 36011 Poltava, Ukraine; 3 Department of Fundamental Disciplines and Informatics, Shupyk National Healthcare University of Ukraine, 04112 Kyiv, Ukraine; 4 Department of Nephrology, Lithuanian University of Health Sciences, LT-44307 Kaunas, Lithuania

**Keywords:** atherosclerosis, noncommunicable diseases, mitochondrial dysfunction, mitochondrion

## Abstract

**Background/Objectives**: This review has been prepared to promote interest in the interdisciplinary study of mitochondrial dysfunction (MD) and atherosclerosis. This review aims to describe the state of this problem and indicate the direction for further implementation of this knowledge in clinical medicine. **Methods**: Extensive research of the literature was implemented to elucidate the role of the molecular mechanisms of MD in the pathogenesis of atherosclerosis. **Results**: A view on the pathogenesis of atherosclerosis through the prism of knowledge about MD is presented. MD is the cause and primary mechanism of the onset and progression of atherosclerosis. It is proposed that this problem be considered in the context of a continuum. **Conclusions**: MD and atherosclerosis are united by common molecular mechanisms of pathogenesis. Knowledge of MD should be used to argue for a healthy lifestyle as the primary way to prevent atherosclerosis. The development of new approaches to diagnosing and treating MD in atherosclerosis is an urgent task and challenge for modern science.

## 1. Introduction

Basic science has been trying to uncover “all the secrets” of atherosclerosis for about 100 years, but there is still no complete solution. Atherosclerosis is a proven pathogenetic basis for the occurrence and progression of cardiovascular diseases (CVDs) [1,2,3,4,5].

High annual mortality rates from CVDs worldwide have determined the great social and medical importance of further study of atherosclerosis as a pathogenetic component of many chronic non-communicable diseases (NCDs) [6,7,8,9,10,11,12,13,14,15]. CVDs and NCDs continue to be challenges for the global community and science [7]. Further study of the fundamental mechanisms of pathogenesis in atherosclerosis is of great scientific importance in the search for new, effective approaches to the prevention and treatment of CVDs and NCDs.

Science has made significant progress in studying the molecular mechanisms of the pathogenesis of diseases in the internal organs of the human body. Over the past few decades, scientists have focused on studying mitochondria’s functions as key participants in the metabolic life of cells and their synthetic activity.

The obtained scientific data became the basis for the conclusion that MD is a key mechanism of all NCDs, CVDs, and atherosclerosis. The review [16] describes exactly how NCDs and atherosclerosis risk factors lead to MD. The molecular mechanisms of the occurrence of atherosclerosis due to dysfunction of mitochondria are presented in detail in reviews [17,18,19,20]. The connection between mitochondrial dysfunction and CVDs and NCDs has been proven [21,22,23,24,25,26]. This opens a new direction in the study of atherosclerosis and changes the paradigm of understanding it.

This review was prepared to strengthen interest in the interdisciplinary study of MD and atherosclerosis. Its aim was to describe the state of the problem of studying the molecular mechanisms of MD and atherosclerosis and indicate the direction for further implementation of this knowledge in clinical medicine.

## 2. MD and Atherosclerosis Is the Path to a New Paradigm of Approaches to the Treatment of CVDs

Integrating and rethinking existing scientific data on the molecular mechanisms of the pathogenesis of atherosclerosis and MD is a promising opportunity to optimize the treatment of CVDs and NCDs. Why is this so? According to modern scientific concepts, the basis of the pathogenesis of atherosclerosis is a metabolic pattern of hypercholesterolemia, dyslipidemia, endothelial dysfunction, and chronic inflammation [5,27,28,29]. Therefore, protocols for pharmacological treatment include the prescription of drugs that reduce cholesterol levels in the blood and drugs that expand the narrowed lumens of blood vessels and reduce the manifestations of endothelial dysfunction [30,31,32]. Of course, this is pathogenetically justified if we rely on the previous paradigm of ideas about the pathogenesis of atherosclerosis without taking into account knowledge about MD as the primary source of all these manifestations. Let us look at the pathogenesis of atherosclerosis through the prism of knowledge about the molecular mechanisms of MD. It becomes clear that hypercholesterolemia, dyslipidemia, endothelial dysfunction, and chronic inflammation are the result of pathologically altered mitochondrial functioning. It is the change in the molecular mechanisms of mitochondrial functioning that triggers the pathogenetic pathways into the occurrence and progression of atherosclerosis. Therefore, it is wise to focus treatment on restoring mitochondrial function to eliminate the source of the problem. Modern conventional pharmacotherapy of atherosclerosis is aimed at the investigation, while mitochondrial dysfunction remains without due attention from doctors and without pharmacotherapeutic correction. To change this situation, it is necessary to shift the emphasis and attract more attention from doctors to MD as a crucial universal mechanism of atherosclerosis, CVDs, and NCDs. Justifying the scientific feasibility of this is an essential task for modern biomedical scientists.

## 3. Pathogenetic Connections Between MD and Atherosclerosis

The connection between MD and atherogenesis is a scientifically proven fact [23,33,34]. This connection is a serious reason to consider MD as one of the central mechanisms of atherogenesis. According to modern scientific concepts, the pathogenetic connection between MD and atherosclerosis is based on a metabolic imbalance in the functioning of mitochondria, which arises and is supported by risk factors for atherosclerosis and NCDs. Excessive nutrition without sufficiently long breaks of hunger [16,35,36,37], physical inactivity [16,23,38,39], and the intake of potentially toxic agents (alcohol, components of tobacco smoke, food stabilizers, food preservatives, and other chemicals) [16,40,41] leads to MD. In essence, MD is an adaptive response of mitochondria to the life activity’s unnatural chemical and biological conditions. There is serious scientific evidence for this. Why has this idea not yet received full scientific acceptance in medicine?

Over the past 30 years, molecular biologists and biochemists have contributed significantly to studying the functions of mitochondria in health and disease in the human body. A large body of theoretical knowledge about MD has now been accumulated, which needs further integration into clinical medicine. However, several questions still have no answers and continue to be discussed. For example, science continues to study which mechanisms of MD are the cause of atherosclerosis, and which of these mechanisms are a response to the progression of the process. MD has been described for almost all organs of the human body [23], but systematic ideas about the formation of MD at the level of the whole organism have not yet been developed. In which organs of the human body does MD occur first? What are the dynamics of MD in the body’s organs? There is no scientific description of how MD in some organs affects mitochondrial dysfunction in other organs of the human body. These are essential questions. The answers to these questions will form a holistic understanding of the molecular mechanisms of metabolic interconnection in the human body in the future. This will make it possible to understand why different people have damage to different organs by the atherosclerotic process and different degrees of atherosclerosis spread. This indicates the need to create a new working theoretical concept that systematizes the available data into a single universe. Creating a holistic system of ideas will become a working model that will enable biomedical scientists to develop ideas for the practical application of knowledge about MD in clinical medicine. What aspects of the pathogenetic relationship between MD and atherosclerosis should be included in the concept? To theoretically prove the existence of a cause-and-effect relationship between MD and the occurrence of atherosclerosis, it is necessary to conceptualize the relationship between risk factors for the occurrence of atherosclerosis and CVDs with MD. To implement this, it is essential to describe such pathogenetic chains as “malnutrition → MD → Atherosclerosis”; “hypodynamia → MD → Aatherosclerosis”; and “bad habits → MD → Atherosclerosis”.

### 3.1. Pathogenetic Chain “Malnutrition → MD → Atherosclerosis”

The concept of “malnutrition” as a risk factor for atherosclerosis and CVDs includes excess nutrition with the absence of periods of hunger of sufficient duration; nutrition with deficiencies of vitamins, microelements, lipoproteins, and proteins; entry into the body of food and drink with potentially toxic agents (alcohol, food stabilizers, food preservatives, and other chemicals).

#### 3.1.1. The Influence of Overnutrition with the Absence of Periods of Fasting of Sufficient Duration on the Molecular Mechanisms of MD That Induce Atherosclerosis

Knowledge about the molecular mechanisms of MD during overnutrition radically changes views on the etiological role of “malnutrition” in the development of atherosclerosis. For a long time, dietary fats have been the main target in preventing atherosclerosis [42,43,44]. Modern medicine pays considerable attention to the pathological effect on the metabolism of easily digestible carbohydrates and foods with a high glycemic index [45,46,47,48]. Contemporary data on the life cycle of mitochondria make it possible to place different emphasis on explaining how to eat to avoid the occurrence of atherosclerosis. Overnutrition leads to MD for two main reasons: (1) The constant supply of food substrates into the cell disrupts the processes of mitochondrial dynamics and mitochondrial biogenesis; (2) Excess food substrates lead to excessive generation of protons and reactive oxygen species (ROS) by mitochondria.

Atherosclerosis, CVDs, and NCDs have become more widespread in the context of the “victory of humanity over hunger” because overnutrition, with the absence of periods of hunger sufficient in duration, changes the collective behavior of mitochondria, their dynamics and biogenesis, and prevents the implementation mechanisms of natural selection of mitochondria in the cell. Mitochondria have ring-shaped deoxyribonucleic acid (DNA) and have many similarities with α-proteobacteria. The most significant similarity with α-proteobacteria is the ability of mitochondria to react to food collectively. If sufficient food substrates exist in the cell, mitochondria are in a fragmented state. Under food substrates’ deficiency conditions, mitochondria unite their membranes and merge into a single mitochondrial network. This allows them to evenly distribute the energy substrate among themselves and survive in conditions of hunger. In this case, mitochondria that have membrane defects cannot join this network and die. This is an essential mechanism of natural selection of mitochondria, which allows biogenesis to be maintained only among morphologically correct mitochondria. The final fusion of mitochondria into a single network occurs only under conditions of complete starvation for more than 12 h [16,35,49,50]. Daily fasting from 6 pm to 7 am, ensures adequate cycles of mitochondrial dynamics, mitochondrial biogenesis and the death of mitochondria with morphological defects, and maintains the existence of a healthy pool of mitochondria in cells. If hunger periods are shorter, then mitochondria do not fuse [49]. Suppose frequent meals without long periods of hunger become a person’s lifestyle. In that case, this will gradually lead to a weakening of the pool of mitochondria and a decrease in the number of mitochondria in the cell, leading to MD, and will trigger the molecular mechanisms of the induction of atherosclerosis.

The widespread availability of food products has led to the problem of excess nutrition, overeating, and, as a consequence, atherosclerosis. Mitochondria must metabolize all food substrates that enter the cell. If an excess food substrate enters the cell, it becomes a metabolic load for mitochondria. At the same time, mitochondria are forced to neutralize these “extra substrates” to change their functioning and generate excess protons and ROS [16] in order to “dissipate” excess energy. If overeating is a person’s lifestyle, then over time, this will create an excess of free radicals in his body, oxidative stress, and trigger the molecular mechanisms of atherosclerosis induction.

#### 3.1.2. Impact of Nutrient Deficiencies on the Molecular Mechanisms of MD That Induce Atherosclerosis

Nutritional deficiencies are another problem of malnutrition, which will also indirectly contribute to the occurrence of MD and atherosclerosis. Mitochondria are membrane structures in which complex biochemical reactions occur. For adequate functioning, membrane enzyme systems require a constant adequate supply of microelements, vitamins, proteins, and lipids [51,52]. Nutritional deficiencies of microelements (iron [53,54,55], zinc [56,57], selenium [58,59,60], copper [61,62,63], magnesium [64,65], coenzyme Q10 [66,67]) and vitamins (A [68], B1, B2, B3, B6, B7, B12, B5, B9 [52,69], C [52], E [52], D [52]) form deficiencies in enzyme components and this disrupts the functioning of electron transport systems on the inner mitochondrial membrane. Deficiencies in the supply of amino acids, phospholipids, and fatty acids disrupt the remodeling of mitochondrial membrane structures. This can lead to morphological defects in mitochondrial membranes, disruption of mitochondrial biogenesis, pathological changes in their functions, and death. The mechanisms were detailed in [52,70,71,72,73,74,75,76].

#### 3.1.3. Microbiome and Molecular Mechanisms of MD That Induce Atherosclerosis

The qualitative composition of food affects the state of the intestinal microbiome [77]. Microorganisms of the human internal environment are in close functional connection with the mitochondria of human body cells. Today, mitochondria are beginning to be considered as internal bacteria in the human body’s cells and are called “mitobiota”. The variety of effects that are jointly caused by mitochondria and symbiotic microorganisms has become the basis for the formulation of the concept of “mitobiota” and “microbiota” as two components of a single functional structure that regulates the homeostasis of the host organism through bioenergetic, epigenetic, metabolic, endocrine, immune, and neurohumoral communications [78,79,80,81,82]. For example, biologically active low-molecular compounds produced by symbiotic microbiota and cellular mitochondria representatives can interact with similar cellular receptors of different tissues [16,83]. Bacterial and mitochondrial DNA can be incorporated into the host nuclear genome [84,85,86,87]. Excessive generation of ROS by mitochondria can interfere with the structure of the intestinal microbiocenosis and damage the integrity of the epithelial barrier of the gastrointestinal tract [88,89,90]. It is currently believed that the overproduction of free radicals and systemic chronic inflammation is the result of the general effects of mitochondria and microbiota [91,92,93,94,95]. Mitobiota and microbiota are considered part of a single system that supplies most endogenous enzymes, substrates, cofactors, and regulators involved in the epigenetic processes of mitochondria, cellular chromatin, and symbiotic and pathogenic microorganisms. For example, the regulation of mitochondrial functions in different tissues of the human body occurs due to the expression of nuclear genes of the mitobiota. It is supported by the additional entry of genetic information into the mitochondria from the microgenome on the human skin and mucous membranes [96,97,98]. Symbiotic microbiota and mitochondria jointly provide energy synthesis and epigenetic modification of the microbial and mitochondrial genome by modeling chromatin, DNA methylation, and microRNA expression [99]. It has been proven that the ability of the microbiota to form such key microbial metabolites, such as short-chain fatty acids, lactate, urolithins, hydrogen sulfide, etc., is important for the functioning of mitochondria [81]. Thus, the intestinal microbiota’s qualitative and quantitative composition affects mitochondrial functions [100,101,102,103,104,105,106]. Therefore, a properly balanced, high-quality food composition with an adequate content of components with probiotic activity (dietary fiber/fiber, hard-to-digest carbohydrates, lactic acid products, etc.) is an essential aspect of a healthy lifestyle, which ensures the preservation of mitochondrial functions. Eating foods that contain stabilizers and preservatives has a detrimental effect on the state of the intestinal microbiome [107,108,109]. This may be an additional pathogenetic component that contributes to the occurrence of MD and the initiation of atherogenesis.

#### 3.1.4. The Influence of Toxic and Potentially Toxic Food Components on the Molecular Mechanisms of MD That Induce Atherosclerosis

Chemical agents can have a direct damaging effect on mitochondria. The specific manifestations of MD are determined by the individual characteristics of the tissues exposed to toxic effects and the type of mitochondrial damage in these tissues. Universal “targets” for exotoxic effects can be [78,110,111] such components of mitochondria as electron transport chains of tissue respiration, enzymes in the oxidative phosphorylation system, membrane components of mitochondria, and mitochondrial nucleic acids. The resulting manifestations and consequences of MD can differ depending on the chemical characteristics of the substances and the specifics of their effect on various tissues of the body.

Alcohol consumption is a classic example of the intake of toxic substances via food and drink [112,113]. Although there is evidence of the anti-atherosclerotic effect of alcohol [114], its effect on mitochondrial function is negative. Ethanol leads to a decrease in cellular protective mechanisms [112,113], an increase in the formation of ROS, and an increase in oxidative stress [112,113,114,115,116,117,118]. Mitochondria components are targets for the action of ROS. This leads to changes in the mitochondrial control of oxidative stress, disturbances in cellular energy metabolism, and processes that control the occurrence and progression of the cell death response [112,119]. Ethanol leads to disruption of the transmission of metabolic signals in tissues, disruption of the function of immune cells, and increased oxidative stress. This contributes to the development of mitochondrial dysfunction. Therefore, ethanol consumption increases the body’s unwanted toxic metabolic load, leading to impaired immune responses and MD. Alcohol-induced MD is a critical contributor to tissue damage [113].

Thus, nutritional disorders are the most significant factor in the development of MD. Daily absence of six-hour meals leads to the degradation of the mitochondrial pool. Overeating and consumption of toxic and potentially toxic substances cause an increase in the generation of reactive oxygen species and oxidative stress, which initiates atherogenesis. A healthy diet prevents the emergence of the pathogenetic chain “Malnutrition → MD → Atherosclerosis” and should be considered as the basis for solving the problems of MD, atherosclerosis, CVDs, and NCDs.

### 3.2. Pathogenetic Chain “Physical Inactivity → MD → Atherosclerosis”

It is generally accepted that metabolic disorders play an important role in the development of atherosclerosis [45,46,47,48]. In the body’s glucose homeostasis, the mitochondria of skeletal muscle tissue play an important role by increasing the clearance of glucose in the blood in response to insulin [120]. Due to the peculiarities of their structure, all muscles, primarily skeletal ones, perform a bioenergetic function at the organismal level. They metabolize food energy through mitochondrial processes into other types of energy (mechanical, electromagnetic, thermal, acoustic, etc.), adenosine triphosphate (ATP), and glycogen [121,122,123]. Therefore, the amount of muscle tissue, the number of mitochondria in its cells, and the morphological and functional state of these mitochondria are fundamentally important for the human body to be in a state of metabolic health and adequate implementation of the phenomenon of life at the quantum level. The indicator of relative muscle content in the body has clinical diagnostic value and can be used to assess mitochondrial functions and metabolism [123] indirectly. The significance of the processes of mitochondrial muscle metabolism is emphasized because about 65 kg of ATP is produced and processed daily in the adult body. In human cardiac muscle, mitochondria comprise about 25–30% of cell volume and consume >6 kg of ATP per day [25,26,124,125].

Regular physical activity supports mitochondrial biogenesis, stimulates physiological intracellular signaling pathways that control mitochondrial functions, and helps increase their number [16]. Regular physical exercise is an important factor in counteracting the processes of mitochondrial degradation. This explains the well-known fact that athletes have a better quality of health and are less prone to MD, atherosclerosis, CVDs, and NCDs [126].

Physical inactivity reduces energy expenditure through mechanical movement. This triggers the pathways of hypothalamic autonomic regulation and gradually leads to a decrease in muscle strength, muscle wasting, and a change in autonomic regulation with the formation of asthenic syndrome [127,128,129,130,131,132].

Therefore, regular physical activity prevents the occurrence of the pathogenetic chain “Physical inactivity → MD → Atherosclerosis.” Regular physical activity should be considered an important basic component in addressing the problems of MD, atherosclerosis, CVDs and NCDs.

### 3.3. Pathogenetic Chain “Bad Habits → MD → Atherosclerosis”

Bad habits such as alcohol abuse and tobacco use increase the exotoxic effect on mitochondria and contribute to the occurrence of MD.

#### 3.3.1. Pathogenetic Chain “Alcohol Abuse → MD → Atherosclerosis”

As noted earlier, alcohol enhances the generation of reactive oxygen species, leads to oxidative stress and dysregulation processes of the immune system, and promotes the initiation of cell death processes [112,113,114,115,116,117,118].

#### 3.3.2. Pathogenetic Chain “Smoking → MD → Atherosclerosis”

Tobacco smoking has a proven cause-and-effect relationship with atherosclerosis [133]. Cigarette smoke contains over 4000 chemicals [134], which have an exotoxic effect on the human body. It has been proven that cigarette smoke in the human body leads to the activation of atherogenesis mechanisms and causes thrombosis, insulin resistance, dyslipidemia, vascular inflammation, abnormal vascular growth and angiogenesis, and loss of homeostatic and regenerative functions of the endothelium [133,135,136,137]. It is now clear that the pathophysiological mechanisms by which tobacco smoke contributes to the occurrence of atherosclerosis, CVDs, and NCDs are also associated with the molecular mechanisms of MD [138,139].

Components of cigarette smoke disrupt the functioning of the mitochondrial enzyme system and oxidative phosphorylation processes. It has been established that cigarette smoke disrupts the processes of oxidative phosphorylation in cardiomyocytes [140,141,142,143] and platelets [144], reduces the activity of myocardial cytochrome oxidase (complex IV [141], leads to an increase in the production of reactive oxygen species by mitochondria [144], reduces the activity of cytochrome oxidase, increases the content of mitochondrial protein ATPase F1, reduces the level of coenzyme Q10 [145,146], causes significant damage to aortic mitochondrial DNA, decreases adenosine nucleotide translocase activity, and increased nitration and inactivation of superoxide dismutase 2 [147]. When combined with hypercholesterolemia, tobacco smoke synergistically increases mitochondrial damage and atherogenesis [147]. Benzo-a-pyrene from tobacco smoke induces atherogenesis [148] and cell death due to necrosis [149].

Cigarette smoke disrupts almost all mitochondrial functions [145,149,150,151,152,153,154,155,156,157,158], increases the generation of ROS [159,160,161,162,163,164], damages mitochondrial DNA and reduces the number of its copies [145,164,165,166,167,168,169,170], disrupts the processes of mitochondrial biogenesis and natural selection [171], induces apoptosis, and alters the signaling redox system of mitochondria [165].

These effects of tobacco smoke have a systemic influence on the mitochondria of the human body. This is confirmed by the presence of a proven cause-and-effect relationship between tobacco smoking and the occurrence of MD and other NCDs as well. For example, a connection has been proven between diabetes mellitus [172,173,174,175,176,177,178,179], cancer [180,181,182,183,184,185,186,187,188,189,190,191,192,193], and a negative effect on the reproductive system of the body [194,195,196,197,198,199].

Consumption of alcohol, other toxic substances, and smoking cause an increase in the generation of reactive oxygen species and oxidative stress, which stimulates atherogenesis. Bad habits cause the emergence of the pathogenetic chain “Bad habits → MD → Atherosclerosis”. They have a systemic negative effect on mitochondria and, therefore, potentiate the mechanisms of MD throughout the body. Giving up bad habits should be considered as an essential component in solving the problems of MD, atherosclerosis, CVDs, and NCDs.

Thus, the cause-and-effect relationship between risk factors for atherosclerosis, CVDs, NCDs, and mitochondrial dysfunction has been proven. Malnutrition, physical inactivity, and bad habits as risk factors for atherosclerosis, CVDs, and NCDs mutually aggravate the pathogenetic effect on mitochondrial function. They cause MD by disrupting all key mitochondrial functions. This creates a pathogenetic background for the further implementation of the molecular mechanisms of MD, which induce atherogenesis.

## 4. Molecular Mechanisms of MD and Atherosclerosis

MD is a universal mechanism for the occurrence and progression of atherosclerosis, CVDs, and other NCDs. What molecular mechanisms of MD link it to atherosclerosis? How do these mechanisms extrapolate to existing knowledge about the pathogenesis of atherosclerosis? How are these mechanisms related to each other and implemented during the progression of atherosclerosis? These are important questions for clinical medicine. To obtain answers to these questions, combining knowledge of molecular biology and clinical medicine is necessary.

### 4.1. Pathological Generation of ROS, Oxidative Stress, and Atherosclerosis

Pathologically increased generation of ROS is a key molecular mechanism of MD that leads to atherosclerosis. ROS are typically signaling molecules produced in mitochondria [200]. The following sources of ROS were identified [201,202]: complexes I and III of the mitochondrial respiratory chain; the monoamoxidase enzyme family and the protein growth factor adapter p66Shc; and nicotinamyl adenine dinucleotide phosphate oxidase 4. The ATP-sensitive potassium channel is a mitochondrial regulator of ROS generation [203]. The state of mitochondrial membranes affects the level of generation of ROS. For example, depolarization of the mitochondrial membrane activates the generation of ROS by increasing the activity of complexes I and III [204]. When overeating in conditions of excess food substrates, hyperpolarization of the mitochondrial membrane occurs due to increased generation of protons, which also leads to increased ROS generation [202,204].

Existing for a long time under the influence of “risk factors of atherosclerosis”, “Unhealthy lifestyle” leads to a pathological increase in the generation of ROS, depletion of antioxidant defense mechanisms, oxidative stress, peroxidation of lipids, proteins, nucleic acids, and cell death [129,205,206]. Oxidized lipoproteins accumulate in the vascular wall and stimulate atherogenesis [207]. Oxidation of proteins and nucleic acids leads to changes in the functioning of mitochondria, the disruption of processes in the tricarboxylic acid cycle, and oxidative phosphorylation [129,205,206].

ROS, as signaling molecules, are involved in the induction of the inflammatory process [203,208]. They are signal transduction molecules that trigger the release of inflammatory cytokines. The consequence of this is the occurrence of endothelial dysfunction and inflammation in the vascular wall [207]. Thus, the molecular mechanism of the pathological increase in the generation of ROS combines the implementation of oxidative stress and the inflammatory theory of atherogenesis. Therefore, mitochondrial oxidative stress predetermines the development of endothelial dysfunction and modulates the inflammatory response, differentiation, growth, apoptosis of endothelial cells, and changes in vascular tone [209].

The insidiousness of the pathological generation of ROS is that a pathological vicious circle arises [202,210,211]: mitochondria generate more reactive oxygen species → reactive oxygen species damage mitochondria → damaged mitochondria generate more reactive oxygen species and other pathological signals that change metabolism, etc. Therefore, the pathological enhancement mechanism of ROS generation can rightfully be considered one that leads to atherosclerosis and supports its progression.

### 4.2. Mitochondrial Lipoprotein Synthesis and Atherosclerosis

Dyslipoproteinemia and hypercholesterolemia are proven key components of the pathogenesis of atherosclerosis [212,213,214]. They are manifestations of a metabolic pattern that occurs long before the clinical manifestations of atherosclerosis and CVDs [212,213,214]. High-density lipoproteins are signal transducers, model inflammatory processes, promoters of immune reactions, and have antioxidant properties [215,216].

Mitochondria are the main synthetic and energy centers of cells [50]. Therefore, they are involved in the synthesis of all components of lipoproteins. For example, high-density lipoproteins and most apolipoproteins are synthesized in hepatocytes. The liver is the body’s central organ of synthesis and metabolism [217]. Enterocytes play a significant role in the metabolism and transport of lipids. Synthesis of apoprotein 1 occurs in the intestine [218]. Accordingly, the occurrence of dyslipoproteinemia should be related to mitochondrial function. It is logical to believe that overeating and the consumption of toxic substances in food will primarily cause a pathological increase in the generation of ROS and MD in the cells of the gastrointestinal tract and liver. In response, a signal transduction change occurs in the hepatocytes’ mitochondria, which gradually leads to dyslipoproteinemia and hypercholesterolemia. This hypothesis requires further study. However, well-known clinical data confirm its viability: atherosclerosis and CVDs always arise gradually against the background of the pathology of lipid metabolism, and chronic diseases of the gastrointestinal tract, gall bladder, and/or liver [219].

With further progression of the pathology, there is an interaction between the mechanisms of pathological enhancement of the generation of ROS and dyslipidemia. This leads to lipid peroxidation by forming an oxidized modification of low-density lipoproteins: malonaldehyde attaches to apoB, and the phospholipid shell is rearranged [220]. These processes occur in the subendothelial space of the arterial wall in the presence of oxidation products of macrophages, foam cells T-lymphocytes, and transition metals (copper, iron) [221]. In the future, inflammation processes can be induced through mitochondrial mechanisms [203,207] and through immune reactions [222,223,224]. For example, inflammation is provoked by cholesterol crystals secreted by foam cells [220], etc.

It has been proven that autoantibodies to low-density lipoproteins can be formed [225,226], which are markers and clinical predictors of atherogenesis [220]. This is consistent with the mechanisms of MD and inflammation. Mitochondria are involved in regulating and implementing immune reactions at the cellular level. Therefore, their participation in autoimmune reactions in atherosclerosis cannot be ruled out. The issue requires further study.

### 4.3. MD and Endothelial Dysfunction

Endothelial dysfunction is an early predictor of atherosclerosis and is closely related to the pathogenesis of atherosclerosis and CVDs [227,228,229,230].

Endothelial dysfunction has been proven to be associated with MD and occurs due to the pathological generation of ROS and oxidative stress [229,230]. Oxidative stress causes inhibition of endothelial nitric oxide synthase. This additionally stimulates the generation of reactive oxygen species by the mitochondria of endothelial cells. At the same time, endothelial cells are affected by other internal and external damaging factors. Recruitment and adhesion of immune cells leads to activation of endothelial cells: inflammation is initiated in the subendothelial space; the endothelium becomes dysfunctional; lipids penetrate the arterial wall due to a decrease in the barrier function of the endothelium [202]. Thus, MD and endothelial dysfunction are related pathological processes in atherogenesis.

### 4.4. Mitochondrial DNA Mutations and Atherosclerosis

Mitochondrial DNA is a good target for oxidative damage because it does not have histones and is poorly repaired [231]. Mitochondrial DNA mutations under the influence of pathological concentrations of ROS and activated peroxidation are important mechanisms for the occurrence and progression of MD, atherosclerosis, CVDs, and other NCDs [202,232,233]. This is so because mitochondrial DNA encodes essential proteins for oxidative phosphorylation and provides information for ribosomal and transport ribonucleic acids [234]. Accordingly, the consequences of damage to mitochondrial DNA are a disruption/reduction in the efficiency of oxidative phosphorylation, a decrease in ATP production, dysfunction of transport and ribosomal nucleic acids, and disturbances in protein synthesis [235].

The cause-and-effect relationship of DNA mutations with all stages of the pathogenesis of atherosclerosis has been proven [236]. A cause-and-effect relationship has been established between mitochondrial DNA mutations and proatherogenic processes: inflammation, aging, and cell apoptosis [237,238,239]. Endothelial cells of blood vessels and circulating immune cells are typical localizations for this pathology [202].

The changes in the functioning of mitochondrial DNA lead to MD. Therefore, mitochondrial DNA copy number-testing has been proposed as a surrogate marker for MD [240]. For example, in a study [241], the study of changes in the number of copies of mitochondrial DNA in CD14+ monocytes provided evidence of mitochondrial dysfunction. This fact confirms the direct involvement of mitochondria in the disruption of the inflammatory response of monocytes [241] and the existence of a causal relationship between dysfunction of mitochondrial DNA, mitochondrial dysfunction, systemic inflammation, and atherosclerosis.

A list of mitochondrial DNA mutations involved in the pathogenesis of atherosclerosis has been established [242].

Mitochondrial genetic variability is an important and scientifically proven factor in the pathogenesis of atherosclerosis [243].

Thus, mutations in mitochondrial DNA cause mitochondrial dysfunction and are associated with pathological processes in atherogenesis.

### 4.5. MD, Chronic Inflammation, and Atherosclerosis

All stages of atherosclerosis are associated with a chronic inflammatory process in the vascular wall [202,244,245]. At the early stage of atherosclerosis, the adhesion of circulating monocytes to activated endothelial cells occurs [202,244,245,246]. Vascular damage occurs, which is accompanied by the expression of adhesion molecules. Further association of monocytes and blood lymphocytes with endothelial adhesion molecules causes the migration of these cells into the subendothelial space of blood vessels with further differentiation [247]. ROS stimulates the release of pro-inflammatory cytokines and chemokines [203]. More and more immune cells enter the *locus morbi*, and the immune response continues. Furthermore, in the vascular wall, under the influence of factors secreted by the endothelium, differentiation of monocytes into macrophages occurs.

Differentiated macrophages phagocytose lipids and transform them into foam cells. They continue to support the inflammatory response and release various pro-inflammatory factors into the plaque environment [248]. The nuclear factor-κB pathway is the cellular response mechanism for releasing pro-inflammatory cytokines. It affects the level of reactive oxygen species in the cell and regulates the expression of genes for adhesion molecules, growth factors, pro-inflammatory cytokines, and inducible enzymes (cyclooxygenase and nitric oxide synthase) [249]. Pro-inflammatory cytokines are also released by mast cells, penetrating the arterial wall. This regulates extracellular matrix production, promotes smooth muscle-cell proliferation, and activates metalloproteinases [250]. Given the role of mitochondria in regulating inflammatory processes, the molecular mechanisms of MD are likely to mediate multiple aspects of local and systemic inflammation during the formation, development, and degradation of atherosclerotic plaque. MD is a source of oxidative stress, pro-inflammatory agents, and molecular mechanisms of cell and tissue damage.

### 4.6. MD, Cellular Energy Deficiency, and Atherosclerosis

At different stages of the pathological process, MD can begin to manifest itself through molecular mechanisms of tissue respiration and oxidative phosphorylation. This results in a disruption in the metabolism of substrates and a decrease in ATP production. Increasing intracellular ATP deficiency leads to the inhibition of energy-dependent processes in the cell and a further aggravation of the mechanisms of tissue respiration and oxidative phosphorylation. This forms another vicious circle caused by cellular energy deficiency. Cellular energy deficiency subsequently leads to changes in the membrane potential of mitochondria and all cell membrane structures. Disturbances in mitochondrial biogenesis will initiate mitochondrial molecular pathological signaling with stimulation of the inflammatory process, histological changes, mechanisms of aging, and cell death [129,205,206]. This is a well-known classical mechanism of MD, which is of fundamental importance for progressing pathology.

### 4.7. MD, Aging, and Atherosclerosis

Atherosclerotic lesions increase with age. With aging, the threshold for susceptibility to atherosclerosis decreases due to disruption of the redox balance mechanism [248,249,250,251,252,253] and MD [254]. The morphology of mitochondria can change during the aging of the body [254] and contribute to the emergence of age-related pathologies [255]. The molecular mechanisms of MD that can cause cellular aging and atherosclerosis include mutations and damage to mitochondrial DNA, mitophagy disorders, changes in energy metabolism, interleukin-6 release, and pathological generation of reactive oxygen species [256]. MD may be exacerbated in senescent cells due to the secretory phenotype associated with aging and other reactive molecules [257,258,259]. Atherosclerosis, aging, and MD are interconnected and mutually potentiate each other. In this case, vicious circles are formed from the molecular mechanisms of MD [255]. Mitochondrial function is impaired with age and is characterized by an increase in mitochondrial DNA mutations, a decrease in the intensity of tissue respiration in the Krebs cycle, a decrease in ATP production, various disorders of the synthetic function of mitochondria, and disorders of mitochondrial mitophagy [260,261,262,263]. This increases the risk of increased progression of atherosclerosis in people as they age.

**Figure 1:**
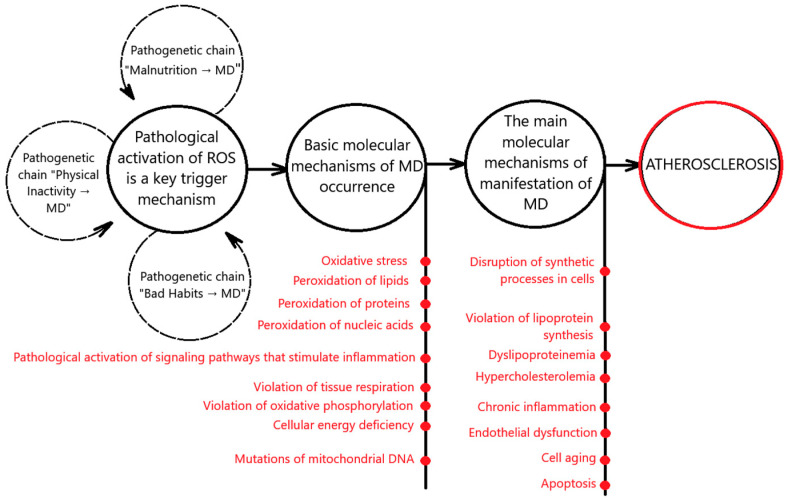
Mechanisms of MD in the pathogenesis of atherosclerosis.

Thus, the main molecular mechanisms of MD that lead to the occurrence and progression of atherosclerosis are pathological activation of the generation of reactive oxygen species and oxidative stress; the violation of lipoprotein synthesis; the pathological activation of signaling pathways that stimulate inflammation, cell aging, and apoptosis; violation of tissue respiration, oxidative phosphorylation, and the occurrence of cellular energy deficiency; and mutations of mitochondrial DNA and the disruption of synthetic processes in cells. MD and atherosclerosis are also associated with mechanisms of cellular aging. This is summarized graphically in Figure 1.

The presented scheme demonstrates the essence and cause-and-effect relationship of the processes occurring but does not reveal the features of their development in the dynamics of pathology progression. For a more complete understanding of the relationship of the molecular mechanisms of MD with the pathogenesis of atherosclerosis, their consideration is required during the catamnesis of the disease.

## 5. Problems of Studying the Progression of MD and Atherosclerosis During the Follow-Up of the Disease

MD and atherosclerosis are characterized by gradual progression during the disease. This is accompanied by varying degrees of MD in different organs at different stages of atherosclerosis. However, there are no models for describing this in modern fundamental science. In what sequence does MD occur in the cells of organs and organ systems during the progression of atherosclerosis? Is there a pattern and logic in the influence of MD in some organs on MD in other organs during atherosclerosis? Answers to these questions will be obtained after full disclosure of all pathogenetic mechanisms of atherosclerosis and MD in all organs in the future. In the meantime, working models of the CVDs continuum [264,265] and the NCDs continuum can be used to explore this issue further [16,205,265,266,267,268,269,270].

**Figure 2:**
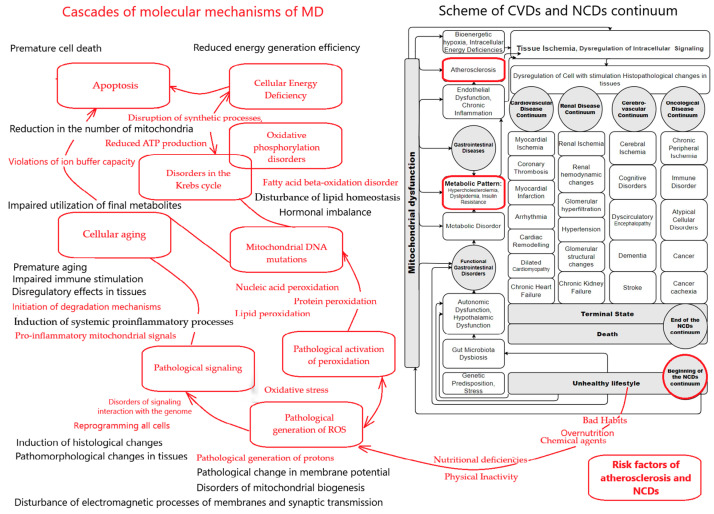
Scheme of key mechanisms of MD in the pathogenesis of atherosclerosis in the context of the NCDs continuum. Note: Cascades of molecular mechanisms of MD are depicted in red on the left side of the figure. The scheme of the CVDs continuum [264,265] and the continua of damage to other internal organs in NCDs [16] are depicted in black on the right side of the figure.

These models describe the development process of pathology in the form of a sequential chain of development of combined pathological conditions, which are characterized by gradual progression, the emergence of a natural sequence of manifestations of the disease, its change over time, and its addition to other pathological disorders. That is, it is a model for representing the development of pathology over time. Figure 2 presents a working model of the overall NCDs continuum [16], which in this review was expanded to include molecular mechanisms of mitochondrial dysfunction and atheroselerosis.

The presented working model describes the connections between the mechanisms of MD and atherosclerosis in a single concept. Simplified, this conceptual model can be described as follows. The CVDs continuum’s scheme by V. Dzau and E. Braunwald [264,265] was the basis for creating a working model for describing the systemic connections of the development of pathology in the main groups of NCDs in the whole organism, taking into account the role of mitochondrial dysfunction [16]. This scheme is on the right side of Figure 2 and demonstrates the stages of the pathology development catamnesis and the connection with MD.

At the beginning of the continuum are risk factors of atherosclerosis, CVDs, and NCDs/unhealthy lifestyles. They cause MD of the gastrointestinal tract and progression of the disorder of their function, which serves as the basis for developing dyslipidemia and metabolic patterns. The following shows the chain of pathogenetic mechanisms leading to atherosclerosis and the place of atherosclerosis in the pathogenesis of NCDs. MD provides the molecular mechanisms of the pathogenesis of atherosclerosis. Atherosclerosis is complexly associated with disturbances of metabolic processes in the human body. Atherosclerosis is part of the profound pathological changes in vascular tissues that lead to dysfunction of organs, vascular complications, and death/end of the disease continuum.

Molecular mechanisms of MD are key at all stages of the development of the disease catamnesis. They are activated under the influence of etiological factors. The main etiological cause of MD and atherosclerosis is an unhealthy lifestyle. Therefore, the risk factors for atherosclerosis and NCDs are in the lower right corner of Figure 1. Bad habits, overeating, chemical agents against the background of hypodynamia, and nutritional deficiencies trigger a cascade of molecular mechanisms of MD. This is shown in the left part of Figure 1. Systematic overeating and the intake of toxic substances into the body leads to the pathological generation of protons on mitochondrial membranes, damage to mitochondrial membrane molecules, disruption of the electromagnetic potential of mitochondrial membranes, and disruption of mitochondrial biogenesis. The consequence of this is the occurrence of pathological generation of ROS by mitochondria. This was described in detail in Section 4.1, Section 4.2 and Section 4.3 of the review. This is a fundamentally important pathogenetic moment that triggers the pathological circle of MD. Firstly, this causes pathological activation of the peroxidation processes of lipids, proteins, and nucleic acids. This also happens in mitochondrial DNA. This causes various disturbances in reactions in the Krebs cycle and oxidative phosphorylation. Consequently, this leads to the gradual emergence of ATP deficiency and disruption of synthesis processes. This gradually leads to energy deficiency and functional disorders in the cells of the corresponding tissues and organs. This was described in detail in Section 4.4 and Section 4.5 of the review. For the prevention of atherosclerosis, CVDs, and NCDs, it is important to understand that they primarily cause MD of the gastrointestinal tract organs and the progression of their dysfunction. This is the basis for the development of dyslipidemia and metabolic patterns [217]. For example, if these processes occur in hepatocytes, then this causes disturbances in lipid synthesis, etc. This is reflected in the right part of Figure 2 as part of the chain of pathogenetic mechanisms leading to atherosclerosis, CVDs, and NCDs. Importantly, the pathological generation of ROS leads to pathological signaling within and between cells. This leads to serious consequences such as cell reprogramming and the induction of histological changes. During the progression of the disease, this causes the appearance of pathological histomorphological to change in tissues. In atherosclerosis, this occurs in the endothelial cells of blood vessels [207,209]. Since this is a universal mechanism, the corresponding pathomorphological changes occur in parallel in the cells of other tissues and organs. This contributes to the emergence of comorbidity and polymorbidity, which are companions of atherosclerosis and manifestations of NCDs. This pathological signaling changes the course of cell metabolic processes, leads to dysregulatory processes, and stimulates inflammation, aging, and apoptosis. Under these conditions, the metabolic processes of the Krebs cycle and oxidative phosphorylation continue to deteriorate, ATP deficiency continues to increase, and the state of general energy deficiency in cells also increases. In cells with a state of energy deficiency, the Krebs cycle and oxidative phosphorylation processes are further disrupted. This closes the “vicious circle” of the pathogenetic influence of the mechanisms of MD on the functional state of cells [16]. Does the localization of cells with MD have conceptual significance for the pathogenesis of atherosclerosis? Of course, it does. MD in tissue cells of the organs of the digestive system leads to disturbances in their functioning, including functional disorders of the digestive organs, their inflammatory processes, and pathological histomorphological changes. The clinical consequence of this is the gradual formation of a metabolic pattern due to disruption of the complex interaction and functioning of the organs of the digestive system. Hypercholesterolemia and dyslipidemia are a consequence of this, an important pathogenetic component of the pathogenesis of atherosclerosis. MD in endothelial cells has an important role in chronic inflammation, endothelial dysfunction, the induction of pathological histomorphological changes, and the formation of clinical and morphological signs of atherosclerosis. In the future, MD becomes a factor that supports the progression of the atherosclerotic process. For example, if a person continues to lead an “unhealthy lifestyle”, then disturbances in the Krebs cycle, oxidative phosphorylation, and pathological pro-inflammatory mitochondrial signaling persist and increase. This leads to a further gradual worsening of cellular energy deficiency, aging, and apoptosis, as well as an increase in the severity of pathological histomorphological changes in an increasing number of cells and tissues of the human body. This is how NCDs gradually begin to form. This was described in detail in Section 4.4 and Section 4.7 of the review. Atherosclerosis is one of the important pathological components that contributes to the progression of NCDs and the development of their complications. For example, the processes of de-stabilization of atherosclerotic plaques are a well-known reason for the occurrence of infarction in organ tissues, which leads to the end of the patient’s disease continuum.

The role of MD in the continuum of atherosclerosis and NCDs is not yet fully understood by science. Research is ongoing, and scientists are working to address these issues.

## 6. Problems in Assessing MD in Atherosclerosis

The problems of studying MD in atherosclerosis are caused by the lack of diagnostic methods for MD in patients with atherosclerosis that are available for widespread use in clinical practice. Existing methods for assessing mitochondrial functions are intended for research purposes, are characterized by high cost and technical complexity, and are focused on scientific experimentation. There are the following methods for assessing mitochondrial functions: methods that are based on the assessment of tissue respiration (the high-resolution respirometry; the Oroboros technology method; the Seahorse technology method; the nuclear magnetic resonance (NMR) technique; the magnetic resonance spectroscopy (MRS) technique); measurements of mitochondrial enzymes; and other methods (for example, examination of metabolomics data to detect metabolic signatures for diagnosis and identification of potential treatment targets, etc.).

### 6.1. Methods for Assessing Tissue Respiration of Mitochondria

To assess tissue respiration using high-resolution respirometry, it is necessary to use sealed reaction chambers and oxygen sensors with high sensitivity and accuracy. This method is technologically complex and requires accurate amperometric measurements of the oxygen consumption rate [271,272].

The Oroboros technology method is based on technology for measuring mitochondrial respiration using a substrate in skeletal muscle. Modular high-resolution respirometry systems are required to perform the study. This allows the study of mitochondria and the measurement of respiration at controlled oxygen levels in combination with redox indicators (co-enzyme Q10 and nicotinamide adenine dinucleotide), assessment of the production of reactive oxygen species, ATP, Ca^2+^, or pH, and mitochondrial membrane potential [23,273,274].

The Seahorse technology method makes it possible to simultaneously study mitochondrial respiration and substrate utilization in skeletal muscles—glycolysis in living cells—in real-time [23,275,276].

The nuclear magnetic resonance (NMR) technique [23,277,278,279] and the magnetic resonance spectroscopy (MRS) technique [23,280,281] are high-tech methods for assessing mitochondrial respiration, which is based on studying the precession of atomic nuclei.

### 6.2. Methods for Measuring Mitochondrial Enzymes

Classical approaches to assessing mitochondrial functions involve measuring of the corresponding oxidative enzymes involved in oxidative phosphorylation (citrate synthase and succinate dehydrogenase) [23,273,274,275,281,282]. Methods for measuring mitochondrial superoxide production [283] exist, including using the MitoSOX probe [284,285,286]. The scientific search for markers to assess mitochondrial function continues.

### 6.3. Other Methods for Measuring Mitochondrial Function

The great promise lies in the future use of metabolomics in large-scale clinical trials. To identify new biomarkers, models are constructed that compare metabolite factors in patients and healthy respondents and identify new specific metabolic pathways, etc. [276].

The technique for indirectly measuring mitochondrial function and metabolic flexibility is based on measuring fat oxidation using indirect calorimetry, stoichiometric equations, and blood lactate levels during exercise. Fats and lactate are important mitochondrial substrates that are metabolized in mitochondria. The method provides an assessment of these parameters during physical activity in patients. An increase in blood lactate levels during exercise indicates a decrease in the ability of mitochondria to oxidize fats—that is, MD [23,280,287,288,289]. This method is of interest in terms of the possibilities of its use when examining a large number of people in an outpatient setting.

An indirect assessment of the metabolic processes of mitochondrial activity is possible by studying the phenomenon of ultraweak photon emission. This phenomenon is explained by the generation of biophotons by DNA molecules and is considered a universal manifestation of the life process [279,280,281,287,288,289,290,291,292,293]. It is well known that all cells have the same set of DNA in their nuclei and a different amount of mitochondrial DNA, which depends on the type of cell and the individual characteristics of biogenesis and mitochondrial dynamics in them. Therefore, the radiation parameters depend to a greater extent on the contribution of mitochondrial DNA, which is explained by the participation of mitochondria in the mechanisms of electromagnetic transmission of genetic information [291]. This issue requires further study. However, biophotons are another viable candidate for assessing mitochondrial functions [294,295]. For example, in studies using the assessment of the level of metabolism of body tissues in vivo using the method of electrophoton emission analysis, qualitative and quantitative differences in biophoton emission indicators were established in functionally healthy respondents and patients with atherosclerosis (with coronary heart disease) [205,295,296]. These preliminary data justify further interest in continuing research in this direction.

## 7. Discussion and Future Perspectives

MD is one of the important scientific problems of modern biology and medicine. However, there is a certain underestimation of its importance among medical scientists. This requires rectifying the situation. This review has been prepared to blur the lines between mitochondrial dysfunction and practitioners. Today, studying MD and atherosclerosis goes beyond biomolecular research and becomes a transdisciplinary problem. Modern knowledge about MD’s role in atherosclerosis occurrence and development should awaken greater interest among medical scientists and pharmacists. Mitochondria are becoming a desirable therapeutic target for preventing and treating atherosclerosis, CVDs, and other NCDs. This is understood by many specialists who continue to deal with this problem [21,22,23,297,298]. The search for new promising pharmacological agents for the correction of MD continues [211,297,298]. New therapeutic approaches using mitochondrial transplantation to replenish or replace damaged mitochondria in the human body continue to be developed [298].

Developing new hypotheses and models of the pathogenesis of atherosclerosis and NCDs is undoubtedly a relevant direction in the study of comorbidity and combination of diseases of internal organs in MD, atherosclerosis, CVDs, and other NCDs. There is a need to study the cause-and-effect relationships between MD in the tissues of different organs. This may provide clues to the causes of comorbidity and combined damage to internal organs in atherosclerosis, CVDs, and NCDs.

A promising direction is the further study of the biophysical mechanisms of mitochondrial functioning associated with their participation in the non-chemical communication of cells. Knowledge about the phenomenon of ultraweak photon emission [276,277,279,280,286,287,288,289,290] and the conceptualized role of mitochondria in the generation of biophotons and the electromagnetic transmission of information from nuclear DNA to other cellular components and between cells [294,295,298] could change views on the pathogenesis of atherosclerosis in the future. Understanding the quantum mechanisms of interaction between molecules and the role of mitochondria in this can open up fundamentally new mechanisms of MD and atherosclerosis.

A significant obstacle to the further integration of knowledge about MD into clinical cardiology and medicine is the lack of routine diagnostic methods for assessing mitochondrial function in an outpatient setting. However, existing trends in the possible use of indirect methods [23,205,280,287,288,289,295,296] give hope that this problem will be solved in the future and that mitochondrial function assessment will become a routine study in medicine.

## 8. Conclusions

Common molecular mechanisms of pathogenesis unite MD and atherosclerosis. The primary molecular mechanism of MD is the pathological generation of reactive oxygen species, which initiates the pathogenetic process and supports its progression.

Modern science has made significant progress in understanding the role of mitochondria in the pathogenesis of atherosclerosis, CVDs, and NCDs. This opens up excellent prospects for forming a new paradigm of approaches to treating these diseases. Although the principles of pharmacotherapy for MD have not yet been developed, the fact of its pathogenetic participation in the development of atherosclerosis cannot be ignored. Knowledge about the role of MD in the development of atherosclerosis should now be used in the argumentation of a healthy lifestyle as the primary way to prevent atherosclerosis, CVDs, and other NCDs. To avoid the occurrence of pathological generation of reactive oxygen species, the following is important: do not have bad habits, do not overeat, take nightly breaks from eating daily for at least 12 h, do not consume alcohol and other potentially toxic substances with food and drink, control the balance of the diet and the intake of adequate doses of nutrients, minerals, vitamins, and probiotic food components. Regular physical activity is also an important method for maintaining the “mitochondrial health” of the body. The development of new approaches to diagnosing and treating MD in atherosclerosis, CVDs, and NCDs is an urgent task and challenge for modern science.

## Data Availability

Not applicable.
